# Factor Structure, Internal Consistency, and Screening Sensitivity of the GARS-2 in a Developmental Disabilities Sample

**DOI:** 10.1155/2016/8243079

**Published:** 2016-02-15

**Authors:** Martin A. Volker, Elissa H. Dua, Christopher Lopata, Marcus L. Thomeer, Jennifer A. Toomey, Audrey M. Smerbeck, Jonathan D. Rodgers, Joshua R. Popkin, Andrew T. Nelson, Gloria K. Lee

**Affiliations:** ^1^Department of Counseling, Educational Psychology, and Special Education, College of Education, Michigan State University, East Lansing, MI 48824-1034, USA; ^2^Department of Counseling, School and Educational Psychology, Graduate School of Education, University at Buffalo, The State University of New York, Buffalo, NY 14260-1000, USA; ^3^Institute for Autism Research, Canisius College, 2001 Main Street, Buffalo, NY 14208, USA; ^4^Summit Educational Resources, 150 Stahl Road, Getzville, NY 14068, USA; ^5^Psychology Department, College of Liberal Arts, Rochester Institute of Technology, Rochester, NY 14230, USA

## Abstract

The Gilliam Autism Rating Scale-Second Edition (GARS-2) is a widely used screening instrument that assists in the identification and diagnosis of autism. The purpose of this study was to examine the factor structure, internal consistency, and screening sensitivity of the GARS-2 using ratings from special education teaching staff for a sample of 240 individuals with autism or other significant developmental disabilities. Exploratory factor analysis yielded a correlated three-factor solution similar to that found in 2005 by Lecavalier for the original GARS. Though the three factors appeared to be reasonably consistent with the intended constructs of the three GARS-2 subscales, the analysis indicated that more than a third of the GARS-2 items were assigned to the wrong subscale. Internal consistency estimates met or exceeded standards for screening and were generally higher than those in previous studies. Screening sensitivity was .65 and specificity was .81 for the Autism Index using a cut score of 85. Based on these findings, recommendations are made for instrument revision.

## 1. Introduction

Autism spectrum disorder (ASD) is characterized by a complex profile of symptoms including social communication impairments and repetitive and restricted interests and behaviors [1]. Although core diagnostic features have been delineated, individuals with ASD exhibit significant variability in their presentation and severity of symptoms. Significant variability in symptom manifestation makes diagnosis complex [2]. Diagnosis is further complicated by the need to differentiate individuals with ASD from individuals with other developmental disabilities that may share some associated features (e.g., language impairment, intellectual disability). Klin et al. [3] noted that “the number of children being referred for developmental disabilities assessments with a differential diagnosis of autism continues to increase each year” (page 772). Given the multiple areas affected, variable symptoms, and challenges involving differential diagnosis, there is a significant need for assessment tools that contribute to accurate diagnosis. This is particularly urgent for individuals with ASD as early diagnosis is essential for access to intensive intervention which has been associated with better long-term outcomes [2].

Measures used in assessments for ASD have been categorized along different dimensions including purpose (i.e., screening versus diagnosis) and level of training needed for administration/scale completion (i.e., professional/trained raters versus untrained raters [4, 5]). At present, two measures recognized for their diagnostic accuracy are the Autism Diagnostic Interview-Revised (ADI-R [6]) and Autism Diagnostic Observation Schedule-Second Edition (ADOS-2 [7]). These measures are the most widely respected diagnostic measures; however they are lengthy to administer and require considerable skill and experience working with individuals with ASD [4, 8]. As a result, the ADOS-2 and ADI-R may be beyond the training of many practitioners and less feasible in some settings (e.g., schools [2, 9]).

Issues with the feasibility (training/expertise requirements, lengthy administration, etc.) of these measures have led to increased reliance on rating scales that can be completed by parents and teachers familiar with the functioning of the individual being assessed. A survey by Allen et al. [9] found rating scales were used far more frequently in ASD assessments by school psychologist than the original ADOS or ADI-R. Although informant rating scales may not be diagnostic on their own, they can contribute important information as part of screenings and/or comprehensive assessments [2, 10] and can assist with differential diagnosis [11]. This is especially useful when the measure assesses multiple constructs, is keyed to a diagnostic framework (e.g.,* DSM-IV or DSM-5*), and provides a quantification of symptoms [3, 5, 8]. Informant rating scales may offer advantages over professionally administered scales (e.g., ADI-R) such as greater efficiency and reduced training. Rating scales also provide normative data, are standardized, and may assist with progress monitoring [4]. Despite their potential, rating scales may have limitations (e.g., lack of alignment between scale items and diagnostic criteria, inadequate psychometric properties) that reduce their validity [4]. These potential problems have led to recommendations for psychometric studies of commonly used rating scales [4, 5].

One of the most commonly used ratings scales is the Gilliam Autism Rating Scale-Second Edition (GARS-2 [12]). A survey published in 2008 found that 40% of school psychologists used the GARS-2 in the majority of their ASD-related assessments [9]. The GARS-2 (a revision of the original scale) is a 42-item informant rating scale designed to assist in the identification and diagnosis of autism and provide information on symptom severity. It can be completed by parents, teachers, and/or clinicians. The items are grouped to form three subscales (Stereotyped Behaviors, 14 items; Social Interaction, 14 items; and Communication, 14 items) and an overall Autism Index. Items were based on definitions of autism delineated at that time in the* DSM-IV-TR* [13] and by the Autism Society of America. For noncommunicative individuals, the Communication subscale is omitted and the Autism Index is calculated based on the other two subscales. While the GARS-2 item content is very similar to the original scale items [5], the test author reported improvements including the rewriting of some items to increase clarity, elimination of the Early Development scale (replaced by a parent interview not included in scoring), renorming based on the 2000 census, and modification of the guidelines for score interpretation [12].

Reliability data are presented in the GARS-2 manual for both internal consistency and stability. Internal consistency estimates were .88 for Social Interaction, .86 for Communication, .84 for Stereotyped Behaviors, and .94 for the Autism Index. Corrected test-retest coefficients (1-week interval) based on parent ratings of 37 children with autism were .88 for Social Interaction, .70 for Communication, .90 for Stereotyped Behavior, and .88 for the overall Autism Index. Validity data were presented based on correlations between subscale items and the subdomain total score. Median values were Social Interaction .55, Communication .53, and Stereotyped Behaviors .53, and the median coefficient for the sum of all items was .47. Sensitivity and specificity were assessed using matched samples of individuals with autism compared to individuals with intellectual disability, multiple disabilities, and typical controls. Using a cut score of 85 for the GARS-2 Autism Index, sensitivity for the autism group was 1.00 versus typical controls, .85 versus the group with intellectual disability, and .84 versus those with multiple disabilities. Specificity values for the same groups were .87, .85, and .84, respectively [12].

Despite the support reported in the manual, concerns have been noted regarding the need for additional details on the standardization sample characteristics, online recruitment, and lack of diagnostic confirmation [14, 15]. Norris and Lecavalier reviewed the GARS-2 and noted other potential problems including the large numbers of children rated by the same teachers (potential response bias) and the similarity between items on the previous version and the GARS-2 which may lead to problems similar to those of the original GARS involving poor sensitivity and specificity [15]. In a study examining the factor structure and reliability of the original GARS using parent (*n* = 241) and teacher (*n* = 119) ratings, Lecavalier found a three-factor solution consisting of Stereotyped Behaviors, Social Interaction impairments, and communication deficits [4], although these appeared generally consistent with the conceptually derived scales of the GARS, nearly half (i.e., 48%) of the items loaded on the stereotyped behavior scale. Most items had an acceptable factor loading on a single factor; however 26% of the items loaded on scales other than that proposed by the test author. Internal consistency (coefficient alpha) was .82 for Stereotyped Behavior, .85 for Social Interaction, and .84 for Communication. Despite all cases in the sample reportedly having ASD, the Autism Quotient score (using the original GARS probability categories) placed nearly two-thirds of the sample in the “below average” probability of autism category or lower, indicating low sensitivity. (Similar concerns regarding the GARS were raised by South and colleagues [16], who reported a screening sensitivity of .48 for the Autism Quotient in their study sample.) Interrater reliability was low (.31–.48) which may have been affected by the use of informants having different roles (parents versus teachers) or other factors. Overall, Lecavalier cautioned that the amount of explained variance in the factor solution was low (i.e., 38%) and recommended caution when using the GARS for screening or diagnosis as many facets of social and communicative impairment were not adequately assessed and several items may only be tapping related features [4].

While prior studies have provided useful psychometric information on the original GARS, the current review yielded only one published psychometric study of the GARS-2. Pandolfi and colleagues conducted an exploratory factor analysis (EFA), as well as a follow-up confirmatory factor analysis (CFA; to determine if the model was replicated) using matched subgroups from the GARS-2 standardization sample [5]. The protocols were completed by parents, educators, and clinicians for individuals reported to have autism. EFA results yielded a four-factor model (accounting for 38.6% of the variance) which consisted of stereotyped/repetitive behaviors, stereotyped/idiosyncratic language, word use problems, and social impairment. Items from the GARS-2 Communication scale were found to be dispersed across the four factors identified in the EFA, with seven items splitting to help create two language/communication-related factors (stereotyped/idiosyncratic language and word use problems). Correlations were moderate between the stereotyped/repetitive behaviors, stereotyped/idiosyncratic language, and social impairment factors. In contrast, the word use problems factor had very weak correlations with the other factors suggesting that it was relatively independent and not autism-specific. The CFA replicated the results and the word use problems factor was again weakly associated with the other factors. Reliability for each scale was above .80 and median item-total correlations were ≥.45. The empirically derived four-factor model led Pandolfi and colleagues to question the clinical utility of the GARS-2 subscales and suggest that the three conceptually derived subscales put forth by the test developer are measuring more than one construct [5]. The researchers suggested that scale/item problems may be a function of some items only describing a correlate of the intended construct and/or the inclusion of complex or “double-barreled” items that assess multiple behaviors or characteristics. Additionally, some items appeared to assess non-autism-specific characteristics which may restrict discrimination between individuals with autism or other developmental disabilities [5].

Given the prominence of rating scales in the assessment of ASD, research is needed to determine their psychometric adequacy. Information in the GARS-2 technical manual details a number of positive attributes; however independent empirical evaluation is limited. Only one study has examined the GARS-2 factor structure and that study raised significant concerns about the validity of the subscales and the measure's clinical utility. Additionally, there have been no published studies evaluating the sensitivity and specificity of the GARS-2 utilizing samples with ASD and other developmental disabilities. Such studies will help establish “cut points that optimize diagnostic accuracy” [5, page 1128]. The prior study of the GARS-2 failed to independently confirm the diagnosis of those in the sample, relied on data from the standardization sample, and did not have data on functional level (e.g., IQ). The studies by Pandolfi and colleagues [5] and Gilliam [12], as well as Lecavalier [4], used ratings by various sources. Use of sources having different roles (e.g., teacher versus parent) may confound results [4]. The purpose of this study was to extend previous research by examining the factor structure, internal consistency, and screening characteristics of the GARS-2 using ratings provided by special education teaching staff for a sample of individuals with ASD and other significant developmental disabilities. Psychometric studies of GARS-2 teaching staff reports are warranted, as teachers and other educational staff members have been identified as being among the most likely individuals to complete the scale in actual practice [12].

## 2. Method

### 2.1. Participants

#### 2.1.1. Developmental Disabilities Sample

Data for the present study were in the form of GARS-2 teacher and agency-staff ratings of 240 communicative individuals with an ASD diagnosis or a diagnosis of another significant developmental disorder. All students were attending a self-contained special education agency for students with ASDs and other significant developmental delays/disabilities in upstate New York. All participants were between the ages of thee and 21 years.

For the overall sample, the mean age was 9.50 (SD = 4.88). Prior test data regarding cognitive skills was extracted from the students' educational and psychological records indicating a mean IQ of 60.61 (SD = 19.61). (The cognitive data were based on a sample of 228 participants. Twelve were deemed untestable due to behavior issues but were considered clearly low functioning based on other available data.) Given the variability in ages, communicative skills, and functional levels, cognitive measures used varied considerably and were not consistent across cases. The gender ratio was approximately 4 : 1 male-to-female (i.e., male = 78.75%, female = 21.25%). The ethnic distribution was 79.58% Caucasian, 15.42% African American, 2.50% Hispanic, 0.4% Native American, and 2.08% unknown. Although socioeconomic status data were unavailable at the individual case level, the agency's records indicated that 31.1% of its students qualified for free and reduced lunch.

Comparisons across ASD and non-ASD subgroups were nonsignificant for all major demographic variables (i.e., IQ *t*[226] = 0.627, *p* = .532; age in years *t*[238] = 0.369, *p* = .712; gender *χ* ^2^(1, *N* = 240) = 1.373, *p* = .241; and ethnicity: analyzed as a 2 × 2 majority versus minority status comparison *χ* ^2^(1, *N* = 235) = 1.874, *p* = .171). (See Table 1 for additional demographic information.)

The ASD subsample consisted of 99 cases diagnosed with autistic disorder and 22 cases diagnosed with Pervasive Developmental Disorder-Not Otherwise Specified (PDD-NOS). ASD diagnoses were made by either a developmental pediatrician, child psychiatrist, or licensed psychologist using* DSM-IV-TR* criteria [13]. Diagnoses for the non-ASD subsample consisted of other developmental disorders and medical conditions (e.g., intellectual disability, severe developmental language disorder, and severe motor impairments). Though such conditions frequently cooccur with ASD, these cases did not meet comorbid ASD criteria.

Importantly, staff psychologists identified 38 cases from the non-ASD condition that had not yet completed an ASD evaluation (to confirm or rule out an ASD diagnosis) at the time of the study. These 38 cases were included in the factor analysis and reliability estimates, but given the diagnostic ambiguity, they were excluded from any discriminant validity comparisons and screening sensitivity/specificity estimates.

#### 2.1.2. Teaching Staff Raters

Ratings of behaviors associated with ASD were required as part of the pretesting for a large program evaluation project. Raters consisted of the primary and extended teaching staff of a large special education agency for students with developmental disabilities in upstate New York. Rating assignments were made by the staff psychologists in consultation with classroom team leaders. The goals of the assignment process were to have each student rated by the staff member who knows her/him best, while also maximizing statistical independence of the cases being rated. Rater familiarity with the student ranged from a minimum of six weeks to a maximum of 28 months (Mo = 4.5 months) of contact. In order to maintain statistical independence across cases, a different teaching staff rater was assigned to each student. This was possible for the majority of available cases, because most classes maintained a 6 : 1 : 1 ratio for instruction and included a substantial classroom team consisting of primary special education teacher, secondary teacher or teaching assistant, classroom aid(s), personal student aid(s), trained volunteer assistant(s), speech pathologist, occupational therapist, physical therapist, and so forth. Typically, if the student had a personal aid, then the personal aid was assigned to be the rater. All ratings were performed over a two-week period in the middle of the school year and were not a part of any diagnostic evaluations.

Though clearly all teaching staff members were aware that each student had a developmental disability, they were not typically aware of whether or not a particular student had a formal diagnosis of autistic disorder or PDD-NOS. However, most teaching staff members were familiar with the general characteristics of ASD, as a result of their special education training or work experience.

### 2.2. Measures

#### 2.2.1. Gilliam Autism Rating Scale-Second Edition

The Gilliam Autism Rating Scale-Second Edition (GARS-2; [12]), a revision of the original GARS [19], is a screening instrument for assessing individuals ages from three to 22 years who exhibit significant clinical and adaptive behavior issues that may reflect symptoms of autism. It is a third-party rating scale intended to be completed by a primary caregiver or someone else who is very familiar with the day to day behavior of the person being rated. It consists of 42 items divided into three subscales with 14 items each. The subscales are named Stereotyped Behaviors, Communication, and Social Interaction. Items are rated on a four-point frequency scale from 0 (*Never Observed*) to 3 (*Frequently Observed*). Each subscale is scored by summing the individual item scores and converting the result into norm-referenced scaled scores with a mean of 10 and standard deviation of 3. The Communication subscale is not completed for cases that are not sufficiently communicative to allow for adequate ratings. Subscale scores are summed and converted to a deviation quotient metric (normative mean of 100 and standard deviation of 15) to form a composite called the Autism Index. This index can be calculated based on the sum of all three subscales for communicative cases or the sum of only the Stereotyped Behaviors and Social Interaction subscales for noncommunicative cases. According to the manual, Autism Index scores ≥85 suggest that autism is “very likely,” scores from 70 to 84 suggest autism is “possibly” present and scores ≤69 suggest that autism is “unlikely” [12, page 31]. A parent interview assessing delays and abnormalities in development during the first three years of life is also included but does not contribute to the GARS-2 scores.

The standardization sample for the test consisted of ratings of 1,107 children and young adults with autism. Raters were recruited through a variety of methods. Sample proportions for geographic region and ethnicity were consistent with census estimates of the general school-age population, while the 4 : 1 male-to-female gender ratio was consistent with expectations based on autism samples in the research literature. Diagnostic confirmation and data regarding cognitive functioning for sample participants were not obtained. The ratio of ratings contributed by professionals versus parents was 8 : 3 for the normative sample. Separate norms are not available by rater type. The manual includes information concerning internal consistency, test-retest reliability, and concurrent validity for the test (see [12]). Pandolfi and colleagues [5] factor-analyzed the standardization sample for the GARS-2 and concluded the relationships among the item responses reflected a four-factor structure.

### 2.3. Procedures

#### 2.3.1. Rating and Data Collection

All data were collected as part of a larger program evaluation project for a large special education agency serving students with ASD and other developmental disabilities. Students ranged in age from three to 21 years and were served in either 6 : 1 : 1 self-contained, 12 : 1 : 1 self-contained, or integrated classrooms. As part of the program evaluation pretesting, the GARS-2 was completed along with three other behavior rating scales. Each staff member completed the four instruments in counterbalanced order. (Results concerning these other instruments will be reported in other forthcoming articles.)

Every attempt was made to assign teaching staff raters who knew each student best, while also maximizing the independence of the ratings across cases. In order to achieve these simultaneous goals, rating responsibilities were distributed across the different types of teaching staff who worked within each classroom (i.e., lead special education teachers, teaching assistants, classroom aids, 1 : 1 personal aids, speech/language pathologists, occupational therapists, and physical therapists). All ratings were performed without access to student records.

A total of 336 students with autistic disorder, PDD-NOS, or other significant developmental disabilities were rated. All ratings took place over a two-week period in the middle of the school year. Completed rating scale packets were immediately reviewed by project research assistants who made sure that all items were completed on each rating scale. These research assistants returned any incomplete rating scales to staff members with instructions (consistent with test manual instructions [12]) for missed item completion.

Each completed rating scale was independently scored by at least two advanced research assistants with graduate level psychometric training, and data from all rating scales were independently double entered into the database by trained research assistants under the supervision of the doctoral level research coordinator.

Based on data supplied by speech/language pathologists who worked with the students, 72 cases were judged to be insufficiently communicative to allow for completion of the 14 Communication subscale items on the GARS-2. Given that complete data on all 42 GARS-2 items were required for inclusion in the factor analysis, these cases were removed. An additional 24 cases were removed due to a lack of rater independence. Thus, the final sample consisted of 240 sufficiently communicative students with ASDs or other developmental disabilities who were independently rated by teaching staff members with the GARS-2.

#### 2.3.2. Data Analyses

Exploratory factor analysis (EFA) was chosen over confirmatory approaches due to the limited literature available concerning the factor structure of the original GARS and GARS-2. The GARS-2 item data were analyzed using principal axis common factor analysis. This particular factor extraction method was chosen because it is considered robust to violations of normality-related assumptions [20]—which were anticipated to be issues for an ASD sample (e.g., most items yielded considerable positive skew).

An additional issue was the ordinal nature of the item scaling. Each of the 42 GARS-2 items is rated on a four-point frequency scale. The discrete nature of the four ordered categories makes the polychoric correlation coefficient, as opposed to the Pearson correlation coefficient, the more appropriate correlation estimation procedure for the matrix of interitem relationships (see [17]). Thus, the 42 × 42 interitem polychoric correlation matrix was used for the EFA.

According to simulations performed by MacCallum and colleagues [21], the sample size required for factor analysis is dependent upon indicator communalities and level of factor saturation. Larger sample sizes are needed when communalities and factor saturation are lower, while smaller samples are likely to yield convergent solutions for the correct number of factors in the presence of high communalities and high factor saturation. Assuming an indicator to factor ratio of 20 : 3 and wide communality (ranging from .20 to .80), simulations demonstrated convergent solutions for the correct factors 100% of the time with samples as small as 60 subjects [21, page 93]. Squared multiple correlation prior communality estimates based on the Pearson correlation matrix ranged from .38 to .81, while maximum correlation prior communality estimates based on the polychoric correlation matrix ranged from .53 to .91. Assuming 42 indicators and three factors, based on the number of GARS-2 items and subscales, the sample size of 240 cases appears more than adequate for assessing the factor structure of the instrument.

The EFA followed the standard factor extraction, rotation, and interpretation phases. In the extraction phase, several strategies were used to determine the number of factors to retain. Parallel analysis [22, 23] was used in conjunction with the Guttman-Kaiser criterion (i.e., eigenvalue >1.00 [24–26]). Initially, a factor was retained if its obtained eigenvalue exceeded the 95th percentile of the random eigenvalue distribution [23] and met the Guttman-Kaiser criterion. Once the number of factors that met these criteria was established, factor solutions consisting of that number of factors ±1 were examined. The most interpretable solution of the three was retained.

Rotation of the factor solution is conducted to enhance the interpretability of the factors. An oblique (promax) rotation was attempted first. If the factors yielded correlations ≥.30, then the obliquely rotated solution was retained. Otherwise, an orthogonal (varimax) rotation was conducted and retained instead.

Each factor was interpreted and named based on what was conceptually common across the items with the highest loadings on it. Factor loadings <.30 were considered nonsubstantive, loadings ≥.30 and <.40 were considered questionable, and loadings ≥.40 were considered substantive. Three of the investigators independently interpreted the factor solutions.

Internal consistency estimates for the factor-based scales and the original GARS-2 subscales were generated using Cronbach's alpha and ordinal alpha [18]. The internal consistency of the Autism Index was calculated using Mosier's 1943 formula for the reliability of a weighted composite [27]. Correlations between the factor-based scales and the original GARS-2 subscales were examined for evidence of convergent and divergent validity. Mean differences between the ASD and non-ASD subsamples were evaluated for all GARS-2 scores. A classification analysis was conducted to evaluate the sensitivity, specificity, positive predictive value, and negative predictive value of the GARS-2 Autism Index's ASD versus non-ASD classifications compared to the prior clinician-determined ASD and non-ASD diagnoses of the cases.

All analyses involving R programming language [28] software packages were run in SPSS 19.0 [29] using the SPSS R menu for ordinal factor analysis [17], which allows users to access and run analyses available through R packages via the SPSS R plugin [30]. The SPSS R menu was used to generate the polychoric correlation matrix, conduct the parallel analysis using the raw item data to generate polychoric correlation matrices, and calculate both Cronbach's alpha and ordinal alpha coefficients. The PAF was conducted in SAS 9.2 [31] using the polychoric correlation matrix exported from R. All other statistical calculations were performed through SAS.

## 3. Results

A principal axis factor analysis (PAF) was conducted in SAS version 9.2 using the polychoric correlation matrix for the 42 GARS-2 items generated by the SPSS R menu for ordinal factor analysis [17]. Prior item communalities were estimated using the maximum correlation method. The number of factors was determined by a combination of parallel analysis with the Guttman-Kaiser criterion and factor interpretability.

The parallel analysis, based on 100 samples, indicated that the obtained eigenvalues from the first three factors exceeded the 95th percentile of the randomly generated eigenvalue distributions. These first three eigenvalues also met the Guttman-Kaiser criterion (i.e., eigenvalue >1.0; first = 18.75, second = 2.59, and third = 2.42) and each accounted for substantial estimates of the common variance (i.e., first = 62.22%, second = 8.58%, and third = 8.03%). (Though an additional three factors also met the Guttman-Kaiser criterion, they did not meet the parallel analysis threshold.)

Based on these preliminary results, solutions consisting of two to four factors (i.e., 3 ± 1) were examined for interpretability. For interpretive purposes, factor loadings <.30 were considered nonsubstantive, ≥.30 <.40 were questionable, and ≥.40 were deemed substantive. The three-factor solution was determined to be most interpretable and will be described in detail. (The two- and four-factor solutions will also be briefly discussed to explain why they were less suitable.)

An oblique (promax, Kappa = 3) rotation was attempted first, given the anticipated correlated structure of ASD symptoms and the conceptual structure of the GARS-2 (i.e., three subscales combined into an overall score). All three factor solutions examined yielded correlated solutions. In the case of the three-factor solution, Factor I correlated .62 with Factor II and .51 with Factor III, while Factor II correlated .47 with Factor III. Thus, the three-factor correlated solution was retained for interpretation.

Both the pattern and structure loading matrices were examined to help interpret the factors—with primary reliance on the pattern matrix. The pattern matrix for the three-factor solution compared to the GARS-2 conceptually derived subscales is given in Table 2. The matrices were examined and interpreted by three of the investigators independently. For the two- and three-factor solutions, factor names assigned across the three investigators were either identical or equivalent in meaning for all factors. Final factor names assigned within the two-, three-, and four-factor solutions were determined by discussion and consensus among the three investigators involved.

### 3.1. Three-Factor Solution

#### 3.1.1. Factor I

Items which loaded highest on this factor were as follows:* whirls, turns in circles; makes rapid lunging, darting movements; flaps hands or fingers; and makes high-pitched sounds (e.g., eee-eee-eee-eee) or other vocalizations for self-stimulation; flicks fingers rapidly in front of eyes*; and so forth. This factor was named Stereotyped and Repetitive Behaviors. Based on the highest factor loading (≥.30 minimum) per item, this factor contained 18 items—including 12 of the 14 items originally located in the GARS-2 Stereotyped Behavior subscale. This factor also includes two items from the GARS-2 Communication subscale and four items from the Social Interaction subscale. In most cases, these additional items either make direct references to repetitive-type behavior (e.g.,* repeats unintelligible sounds*,* does things repetitively*) or could be peripherally related to such behaviors (e.g.,* uses toys inappropriately; laughs, giggles, cries inappropriately; uses gestures instead of speech; and responds negatively to commands*).

#### 3.1.2. Factor II

Factor II was defined primarily by items such as the following:* is unaffectionate; avoids establishing eye contact; withdraws, remains aloof, or acts standoffish in group situations; looks away or avoids looking at speaker when name is called; resists physical contact with others*. This factor was named Social Avoidance and Withdrawal. Based on the highest factor loading (≥.30 minimum) per item, this factor contained 16 items—including 8 of the 14 items originally located in the GARS-2 Social Interaction subscale. However, it also includes six items from the GARS-2 Communication subscale and two items from the Stereotyped Behaviors subscale. The higher-loading items from these other GARS-2 subscales make conceptual sense as expressions of social avoidance or withdrawal (e.g.,* avoids eye contact, looks away when name is called, avoids asking, and fails to initiate conversation*) or problems with the social use of language (e.g.,* speaks with flat affect*) that may repel others involved in the social interaction.

#### 3.1.3. Factor III

This factor was named Atypical Language and Communication. Items loading highest on Factor III were as follows:* uses pronouns inappropriately (e.g., refers to self as “he,” “you,” and “she”); uses the word I inappropriately (e.g., does not say “I” to refer to self); repeats words out of context (i.e., repeats words heard at an earlier time, e.g., repeats words heard more than one minute earlier); repeats words or phases over and over; inappropriately answers questions about a statement or brief story*; and so forth. Based on the highest factor loading (≥.30 minimum) per item, this factor contained 8 items—including six of the 14 items originally located in the GARS-2 Communication subscale. Beyond these core six items, the weak loading item* upset when routines change* (originally from the GARS-2 Social Interaction subscale) may make some conceptual sense as being related to atypical language (e.g., as a result of failing to understand the change or not having sufficient expressive language to communicate displeasure appropriately), but it is not directly associated with language/communication. Any other items that loaded on this factor had loadings in the questionable range (>.30 <.40) and tended to yield such questionable loadings on multiple factors.

#### 3.1.4. Problematic Items

Within the three-factor solution, several items yielded questionable loadings (>.30 <.40). These items are as follows: 4: eat specific foods; 19: responds inappropriately; 23: uses “yes”/”no” inappropriately; 40:* upset when routines change*; 41:* responds negatively to commands*; and 42:* lines up objects*. In addition, items 19, 23, and 42 each yielded questionable loadings on more than one factor. As a result, all six of these items do not clearly and cleanly belong to any factor-based scale.

Most potentially mixed items showed a clear dominant loading on one factor, making item assignment clear (e.g., 11:* prances*). However, item 15 (*repeats [echoes] words*) and item 18 (*speaks with flat affect*) required more discussion and judgment, because in each case the primary loading was moderate and the secondary loading was not far from it and close to the .40 cut for a substantive loading. For both, each item's statistical overlap between the two factors made conceptual sense (i.e., repeating or echoing words is both a type of atypical language use/communication and a type of repetitive behavior; speaking with flat affect is both socially awkward and communicatively atypical). Though revising these items should be considered, there appear to be both statistical and conceptual reasons to retain them and assign each to its primary factor.

### 3.2. Two-Factor Solution

In the two-factor solution, high loading items for Factor I appeared to be predominantly about Social Avoidance and Withdrawal, while high loadings on Factor II primarily reflected language and communication abnormalities. The items from the GARS-2 Stereotyped Behaviors subscale were distributed across the two factors and tended to load in a mixed manner on both of them. (Overall, at least 16 items loaded on both factors.) These results were not clearly interpretable for a large number of items and suggested the need for at least one additional factor.

### 3.3. Four-Factor Solution

The first three factors of the four-factor solution were similar to those of the three-factor solution (Factor I = Social Avoidance and Withdrawal, Factor II = Stereotyped and Repetitive Behaviors, and Factor III = Atypical Language and Communication). The fourth factor consisted of six items:* uses gestures instead of speech*;* repeats unintelligible sounds*;* responds negatively to commands*;* licks or eats inedible objects*;* injures self*; and* laughs, giggles, cries inappropriately*. In general, the items of this factor appeared reflective of lower functioning behaviors, with the first two items suggesting lack of verbal expression, the third item perhaps a result of a lack of understanding spoken language, and the remaining three being tendencies toward pica, self-injurious behavior, and lack of emotional control (e.g., labile emotions/inappropriate affect).

This factor was not well defined and resulted in considerable discussion over how it could be interpreted. In addition, this overall solution included a greater number of items loading on more than one factor than the three-factor solution. Following discussion among those who independently interpreted the factor solutions, it was decided that the fourth factor was not well defined and included a majority of items that would fit reasonably well elsewhere and that the overall four-factor solution included too many items with mixed loadings and was less readily interpretable when compared to the three-factor solution. It was therefore rejected in favor of the three-factor model.

### 3.4. Internal Consistency Estimates

Internal consistency reliability estimates were calculated for each factor, original GARS-2 subscales, and the GARS-2 Autism Index. Estimates based on both Cronbach's alpha and ordinal alpha are reported in Table 3 for the factor-based scales and original GARS-2 subscales. Cronbach's alpha is the better known estimate but tends to be biased low for ordinal, skewed, and non-tau equivalent data [18, 32]. The ordinal alpha estimate is calculated by replacing the Pearson correlations with polychoric correlations in the alpha formula [18]. This estimation procedure corrects alpha for the ordinal and skewed nature of the data. (However, the assumption of tau equivalence is still necessary and, thus, the corrected estimate will still be considered a lower-bound estimate of reliability.) For the factors, Cronbach's alpha estimates ranged from .84 to .92 and ordinal alpha estimates ranged from .89 to .95. For the original three GARS-2 subscales, Cronbach's alpha estimates ranged from .89 to .90 and ordinal alpha estimates ranged from .92 to .94.

Given that the GARS-2 Autism Index is calculated as a linear transformation of the sum of subscale scores and not derived directly from the sum of the item scores, the composite reliability was calculated using the formula provided by Mosier [27]. This formula takes into account the subscale reliability estimates, subscale variances, and the correlations among the subscales. When calculated using Cronbach's alpha estimates for the subscales, the composite reliability was .96. When calculated using ordinal alpha estimates for the subscales, the composite reliability was .97.

### 3.5. Relationships between Factors and GARS-2 Subscales

By examining Table 4, within each row across columns, it is clear that each factor correlates highest, though imperfectly, with its target GARS-2 subscale and correlates relatively lower with the other GARS-2 subscales. This pattern of correlations suggests that the three-factor solution demonstrates some level of consistency with the intended construct pattern reflected in the three original GARS-2 subscales. However, the lack of near perfect convergence reflects important factor versus subscale disagreements over item placement (see Section 4). In addition, an examination of specific items suggests that Factor III likely reflects a more narrow atypical language/communication construct than originally intended for the GARS-2 Communication subscale.

### 3.6. Clinical Validity and Classification Accuracy

As mentioned under Method, 38 cases from the non-ASD group had not yet been evaluated for possible ASD at the time of the study. Because of the potential ambiguous diagnostic status of these cases, they were not included in the following discriminant validity comparisons or classification accuracy analyses. Thus, for the following analyses, the revised overall sample size was 202 cases (*n* = 121 ASD, *n* = 81 non-ASD).

The clinical or discriminant validity of the GARS-2 Autism Index and subscales was assessed via mean comparisons. As expected, the GARS-2 scores for the Autism Index and all the three subscales were significantly higher for the ASD group than the non-ASD group. (See Table 5 for details.)

A classification analysis of the GARS-2 Autism Index was conducted by comparing the known clinician-determined ASD status of cases with the predicted ASD status of cases based on the Autism Index cut score of ≥85 (indicative of a “very likely” probability of autism according to the test manual and the interpretive guide on the cover of the GARS-2 summary/response booklet [12]). For the study sample, the Autism Index classifications yielded a sensitivity of .6529 (indicating accurate test detection of 65.29% of the cases with known formal ASD diagnoses), a specificity of .8148 (indicating 81.48% accuracy in ruling out of ASD among those cases without an ASD diagnosis), positive predictive value of .8404 (indicating that a positive ASD classification by the GARS-2 suggests an 84.04% likelihood of the case actually having a formal ASD diagnosis), and negative predictive value of .6111 (indicating that a negative or non-ASD classification by the GARS-2 suggests a 61.11% likelihood of the case not having a formal ASD diagnosis).

## 4. Discussion

In the context of the study sample of 240 participants with ASDs and other conditions frequently confused with ASDs, the three-factor correlated solution for the GARS-2 was most interpretable. The three factors named Stereotyped and Repetitive Behaviors (Factor I), Social Avoidance and Withdrawal (Factor II), and Atypical Language and Communication (Factor III) showed reasonable correspondence to the three GARS-2 conceptually derived subscales, respectively, named Stereotyped Behaviors, Social Interaction, and Communication. In terms of item overlap, 85.71% of the GARS-2 Stereotyped Behavior subscale items loaded primarily on Factor I (Stereotyped and Repetitive Behaviors), 57.14% of the Social Interaction subscale items loaded primarily on the Factor II (Social Avoidance and Withdrawal), and 42.86% of the Communication subscale items loaded on Factor III (Atypical Language and Communication). Though imperfect, convergent correlations ranged from .87 to .97 between each factor-based scale and its corresponding GARS-2 subscale, with all other factor-to-subscale relationships yielding the expected divergent pattern. Additionally, the correlated nature of the three factors is consistent with a possible higher-order factor reflecting the composite Autism Index. Thus, these data generally support a three-subscale and overall composite conceptualization of the GARS-2.

However, the factor analytic results also suggest a number of concerns that could inform the revision of the instrument. The lack of near perfect convergence between each factor-based scale and its corresponding GARS-2 subscale (see Table 4) reflects the presence of wrongly placed items on the GARS-2 subscales, which contribute construct irrelevant variance to the subscale scores and attenuate the correlation with the intended factor. The pattern matrix indicated that 16 (i.e., 38.10%) of the GARS-2 items did not load on the factor most reflective of the intended subscale but typically loaded on a different factor. Such results suggest that these discrepant items have been assigned to the wrong subscale. Though the GARS-2 was conceptualized with 14 items per subscale, the EFA suggested that a subscale based on Factor I contains 18 items, second subscale based on Factor II has 16 items, and a third subscale based on Factor III contains 8 items. In addition, six items (i.e., 4, 19, 23, 40, 41, and 42) yielded questionable or even low and mixed loadings, which suggested that they do not belong to any of the three factor-based subscales. This pattern of results gives clear direction for potential item-to-scale reassignment and item revisions.

A comparison of this three-factor correlated solution with that of Lecavalier [4] is very instructive. Lecavalier examined the factor structure of the original GARS using principal components analysis. (Differences in item wording between the GARS and GARS-2 for the core 42 items are negligible.) Lecavalier also found a correlated three-factor structure. An examination of the item overlap between the three-factor structures across the two studies indicated that 36 out of 42 items (i.e., 85.71%) loaded on the equivalent factor across both studies. In addition, five of the six items that loaded differently across the two studies (i.e., items 4, 19, 23, 40, and 42) were among the six items determined to be problematic (due to questionable or low and mixed loadings) in the current study. Three of the six discrepant items (i.e., items 19, 40, and 42) also yielded primary loadings below .30 in the Lecavalier results, while still another (i.e., item 4) yielded a questionable range primary loading. (Though it should be noted that the factor analytic approach in the Lecavalier study likely yielded generally lower factor loadings due to the use of Pearson correlations, the level of relative agreement across the two studies on where items fit and which items are problematic is impressive.) The factor structures across the two studies are clearly convergent and virtually identical, even given different samples, different rater types, and different EFA approaches.

A comparison of the correlated three-factor model from the current study with the correlated four-factor solution found by Pandolfi and colleagues [5] using the GARS-2 standardization sample is also instructive. Pandolfi et al.'s Factor I, Stereotyped Behavior (17 items), and Factor IV, Social Impairment (14 items), both line up well with present study's Factor I, Stereotyped and Repetitive Behaviors (18 items), and Factor II, Social Avoidance and Withdrawal (16 items), respectively. The stereotyped behavior factor shares 16 items across the two studies (i.e., 88.88%–94.12% overlap), while the social impairment/avoidance factor shares 12 items across the two studies (i.e., 75%–85.71%). The high level of agreement between the corresponding factors across the two studies strongly indicates that they measure the same, conceptually consistent construct. In contrast, the items from the Atypical Language and Communication factor in the present study split up to form two separate language factors in the Pandolfi et al. study—one reflecting stereotyped/idiosyncratic language and the other assessing word use problems. When the four-factor EFA solution was assessed in the present study, it clearly did not lead to two separate and interpretable language factors. This difference between the factor solutions across the two studies may be a reflection of differences between the cases and/or raters between the present study and the GARS-2 standardization sample. However, the two studies agree on the presence of a factor reflecting stereotyped behavior, another reflecting social impairments, and at least one other language/communication factor. It is also noteworthy that five of the seven items (i.e., items 2, 4, 18, 19, 23, 40, and 41) that loaded differently across the two studies were among the six problematic items noted in the three-factor solution for the present study. Five of these seven items also yielded low to questionable loadings in the Pandolfi et al. four-factor EFA solution. Taken together with the Lecavalier [4] results, several of the same items were identified consistently as problematic across GARS and GARS-2 factor analytic studies.

### 4.1. Internal Consistency Estimates

Both Cronbach's alpha and ordinal alpha estimates were reported in the current study. It is noteworthy that these coefficients were generally higher than those reported in other sources (e.g., [4, 5, 12]), and this was the case regardless of the type of alpha coefficient. Though these estimates clearly meet or exceed reliability standards for screening purposes (see [33]), it is likely that the greater heterogeneity of the present study sample contributed to the relatively higher internal consistency numbers. For example, the Autism Index standard deviation for the total (i.e., combined ASD and non-ASD) sample was 21.20 in the present study, which is higher than that of the GARS-2 standardization sample (SD = 15) and the Autism Quotient standard deviation (SD = 15.4) for the original GARS reported by Lecavalier [4].

### 4.2. Clinical Comparisons

As expected mean comparisons between the ASD and non-ASD groups were statistically significant (*p* < .001) and substantive for all GARS-2 subscales and the Autism Index. The mean Autism Index for the ASD subsample (M = 90.81) indicated that the average ASD case fell within the “very likely” range for autism according to the GARS-2 manual, while the mean score for the non-ASD subsample (M = 70.40) placed the average non-ASD case at the lower end of the autism being “possibly” present range.

The classification accuracy of the GARS-2 Autism Index in classifying the known ASD and non-ASD cases yielded a sensitivity of .6529 and specificity of .8148. The sensitivity estimate is higher than that reported in prior studies with the GARS (e.g., [4, 16]). One factor likely critical to the higher sensitivity is that the original GARS manual recommended a cut score of ≥90 for the Autism Quotient [19], while the GARS-2 manual recommends a cut score of ≥85 for the Autism Index [12]. With a ≥90 cut score, the sensitivity for the present study sample would drop to .5289. This estimate is still higher than the .378 sensitivity reported in the Lecavalier [4] study with the GARS but similar to the .48 sensitivity reported in the South et al. [16] study with the GARS. Other factors to consider are that the teaching staff members who provided the ratings in the present study were very familiar with developmental disabilities, as a result of education and/or direct work experience. Thus, they may be unusually well suited to identify ASD relative to a more typical rater from the general population. However, it is also important to note that the non-ASD comparison cases in the setting for this study tend to share at least some associated features with ASD. Thus, the clinical discrimination context is maximally difficult. Regardless of these various factors, it is still critical to note that the ratings ended up misclassifying 34.71% of the ASD cases as non-ASD—even with the lower Autism Index cut score of ≥85. This suggests that even when completed by special education teaching staff, the GARS-2 would likely miss one-third of the cases with ASD. Thus, it should be used only with caution and clearly not in isolation.

### 4.3. Strengths of the Study

The study involved a number of strengths. First, instruments like the GARS-2 are intended to be used with those suspected of having an ASD. The mixed sample used in this study, including both those with formal ASD diagnoses and those with other significant developmental disabilities, matches well to the actual population and discriminant clinical situation intended for such a measure. Factor analyzing the GARS-2 with such a population is a useful preliminary assessment of the robustness of the factor structure—beyond just those with a known ASD diagnosis. Second, diagnostic and educational records were available for staff psychologists (who were not raters) to verify diagnoses. Third, cognitive data were available for 95% of the sample—with clear indications of lower functional levels for the 12 cases without cognitive test data. Fourth, the use of special education teaching staff only as raters allowed for a more clearly defined rater population, in contrast to prior studies (including the standardization sample for the GARS-2) that combined ratings across professionals and parents. It is noteworthy that the GARS-2 manual suggests that professionals, and not parents, are likely to be the primary GARS-2 raters more often in actual practice [12]. Fifth, steps taken to assure rater independence, scale completeness, and data quality control were very thorough. Sixth, data were factor analyzed using methods appropriate for ordinal item data (i.e., polychoric correlations) and used a factoring method (i.e., PAF) robust to deviations in the item distributions. Finally, the availability of relatively large ASD and non-ASD subsamples allowed for a more comprehensive assessment of the sensitivity, specificity, positive predictive value, and negative predictive value than was possible in most prior studies.

### 4.4. Limitations

Most of the study limitations relate to the generalization of the results. First, though the formal ASD diagnoses likely generalize well to clinical practice, it would have been preferable to have diagnoses independently confirmed using a gold standard diagnostic instrument (e.g., ADI-R [6], ADOS-2 [7]). However, given the lack of a perfectly reliable and valid ASD diagnostic approach, understanding the relationships between a measure and a variety of different diagnostic outcome sources is helpful. Second, all study raters were special education teaching staff with experience working with those with ASD and other significant developmental disabilities. Thus, results may not generalize well to raters without such backgrounds. However, the similarity of the resulting factor structure to that found by Lecavalier [4] in the original GARS suggests at least some preliminary generality across rater types and sample contexts. Third, the 14 items of the Communication subscale can only be completed for cases that are sufficiently communicative. Given that the factor analysis examined the facture structure for all 42 items, results only generalize to communicative cases. Fourth, though also being a strength of the study, the use of the mixed developmental disabilities sample likely increased heterogeneity—which may restrict generalization of some results (e.g., alpha coefficients) to more homogenous populations.

### 4.5. Recommendations for Instrument Revision

Given a review of the GARS-2 manual, available research, and factor analytic findings, the following recommendations are made concerning the revision of the instrument. First, the manual needs a more clearly defined sampling plan and better sample characterization. It is most important that ASD diagnoses be verified and functional levels established. Second, clarify the ratio of raters to ASD cases rated. Ideally, each rater contributes one rating to the normative sample in order to maintain statistical independence. Third, thoroughly explore the development of separate normative tables for different rater types (e.g., parents versus teachers) and for different age groups. Though the manual notes that score correlations with age are low to negligible, this does not necessarily rule out nonlinear age changes or small but potentially important differences. Fourth, strongly consider the reassignment of items to scales based on factor analyses—paying particular attention to those item clusters that appear consistent across different factor analytic studies. Fifth, examine problematic GARS-2 items from the factor analyses. Consider revising or deleting and replacing items with low primary factor loadings, low and mixed loadings, or that including complex wording. Finally, it appears likely that some items yielding low loadings may include content considered important for diagnosing ASDs. In such cases, it is important to assess the wording of the item, whether item revision is reasonable, and to consider whether more items with similar content should be added as the basis for a new factor/subscale.

## Figures and Tables

**Table 1:** Developmental disabilities sample demographic characteristics by subsample and overall.

Demographic characteristic	Autism spectrum disorders *n* = 121	Nonautism spectrum disorders *n* = 119	Overall sample *N* = 240
Age in years	M = 9.39 (SD = 4.58)	M = 9.63 (SD = 5.19)	M = 9.50 (SD = 4.88)
IQ	M = 59.82 (SD = 19.69)	M = 61.45 (SD = 19.57)	M = 60.61 (SD = 19.61)
Gender	Male 81.81% (*n* = 99) Female 18.18% (*n* = 22)	Male 75.63% (*n* = 90) Female 24.37% (*n* = 29)	Male 78.75% (*n* = 189) Female 21.25% (*n* = 51)
Ethnicity			
Caucasian	82.64% (*n* = 100)	76.47% (*n* = 91)	79.58% (*n* = 191)
African American	13.22% (*n* = 16)	17.65% (*n* = 21)	15.42% (*n* = 37)
Hispanic	1.65% (*n* = 2)	3.36% (*n* = 4)	2.50% (*n* = 6)
Native American	0.0% (*n* = 0)	0.84% (*n* = 1)	0.42% (*n* = 1)
Unknown	2.47% (*n* = 3)	1.68% (*n* = 2)	2.08% (*n* = 5)

**Table 2:** Pattern matrix of factor loadings for GARS-2 three-factor solution.

GARS-2 *subscale*/item	Factor I	Factor II	Factor III
*Stereotyped Behaviors*			
(1) Avoids eye contact	.08	***.80***	−.03
(2) Stares	***.59***	.27	.07
(3) Flicks fingers	***.79***	.05	−.08
(4) Eat specific foods	.16	***.34***	.22
(5) Licks or eats inedible objects	***.75***	.15	−.23
(6) Smells or sniffs objects	***.63***	.12	.00
(7) Whirls, turns	***.88***	−.14	.06
(8) Spins objects	***.64***	−.04	.08
(9) Rocks back and forth	***.73***	.10	−.12
(10) Lunging Movements	***.82***	−.10	−.03
(11) Prances	***.73***	−.10	**.30**
(12) Flaps hands or fingers	***.80***	−.04	.00
(13) High-pitched sounds	***.81***	.10	.04
(14) Injures self	***.63***	.11	.09

*Communication*			
(15) Repeats (echoes) words	**.43**	−.10	***.58***
(16) Repeats words out of context	.14	−.03	***.82***
(17) Repeats over and over	.19	−.01	***.73***
(18) Speaks with flat affect	−.07	***.52***	.**39**
(19) Responds inappropriately	**.31**	***.34***	.21
(20) Looks away when name is called	.08	***.77***	.03
(21) Avoids asking	.05	***.70***	−.04
(22) Fails to initiate conversation	.10	***.58***	.14
(23) Uses “yes”/“no” inappropriately	.13	***.31***	**.30**
(24) Uses pronouns inappropriately	−.13	−.09	***.91***
(25) Uses “I” inappropriately	−.16	.03	***.83***
(26) Repeats unintelligible sounds	***.55***	.22	.07
(27) Uses gestures instead of speech	***.44***	**.33**	−.17
(28) Inappropriately answers questions	−.10	.12	***.70***

*Social Interaction*			
(29) Avoids eye contact	.15	***.71***	.02
(30) Stares or looks unhappy	.19	***.67***	−.02
(31) Resists physical contact	−.04	***.74***	−.09
(32) Nonimitative of others	.10	***.68***	−.04
(33) Withdrawn, aloof, standoffish	−.01	***.80***	.09
(34) Unreasonably fearful	.05	***.48***	.15
(35) Unaffectionate	−.24	***.82***	.03
(36) Looks through people	.21	***.70***	−.08
(37) Laughs, giggles, cries inappropriately	***.45***	**.31**	.11
(38) Uses toys/objects inappropriately	***.66***	.11	.09
(39) Does things repetitively	***.55***	.10	**.34**
(40) Upset when routines change	.24	.19	***.32***
(41) Responds negatively to commands	***.38***	.19	.18
(42) Lines up objects	**.30**	.07	***.31***

*Note*. All items are paraphrased to conserve space. Factor loadings ≥.30 are in bold, while the highest factor loading for each item is in italic font.

**Table 3:** Internal consistency estimates for factor-based scales and GARS-2 subscales.

Factor/subscale	Cronbach's alpha	Ordinal alpha	Number of items
Factor I	.92	.95	18
Factor II	.92	.94	16
Factor III	.84	.89	8

Stereotyped Behavior	.90	.94	14
Communication	.89	.92	14
Social Interaction	.89	.92	14

*Note*. Cronbach's and ordinal alpha coefficients are estimated using the SPSS R menu [17]. Ordinal alpha is calculated using polychoric correlation coefficients in the alpha formula [18].

**Table 4:** Correlations between factor-based scales and GARS-2 subscales.

	GARS-2 Stereotyped Behaviors subscale	GARS-2 Social Interaction subscale	GARS-2 Communication subscale
Factor I: Stereotyped and Repetitive Behaviors	***.97***	.81	.73
Factor II: Social Avoidance and Withdrawal	.68	***.91***	.79
Factor III: Atypical Language and Communication	.51	.57	***.87***

*Note*. Raw sums of item scores were used to calculate the factor-based scales and GARS-2 subscale scores. In the case of the factor-based scales, only items with primary loadings ≥.40 were used in the composite. Correlations on the diagonal are convergent and correlations on the off diagonal are divergent.

**Table 5:** GARS-2 means and standard deviations for total sample, adjusted total sample, ASD cases, and non-ASD cases.

GARS-2 subscale/composite	Total sample *N* = 240	Total sample (adjusted) *N* = 202^a^	ASD cases *n* = 121	Non-ASD cases *n* = 81^a^	ASD versus non-ASD comparisons
Stereotyped Behavior	M = 6.71 (SD = 3.58)	M = 6.80 (SD = 3.57)	M = 8.05 (SD = 3.53)	M = 4.93 (SD = 2.74)	*t*(200) = 6.722, *p* < .001^*∗∗∗*^
Communication	M = 8.23 (SD = 3.80)	M = 8.32 (SD = 3.84)	M = 9.70 (SD = 3.48)	M = 6.26 (SD = 3.41)	*t*(200) = 6.943, *p* < .001^*∗∗∗*^
Social Interaction	M = 6.72 (SD = 3.45)	M = 6.76 (SD = 3.46)	M = 7.94 (SD = 3.10)	M = 4.99 (SD = 3.23)	*t*(200) = 6.532, *p* < .001^*∗∗∗*^

Autism Index	M = 82.14 (SD = 21.20)	M = 82.62 (SD = 21.33)	M = 90.81 (SD = 18.94)	M = 70.40 (SD = 18.78)	*t*(200) = 7.533, *p* < .001^*∗∗∗*^

*Note*. GARS-2 subscale scores are in standard score units (normative M = 10, SD = 3) and the Autism Index is standardized according to a deviation quotient metric (normative M = 100, SD = 15).

^a^Thirty-eight cases were removed from the non-ASD condition for this analysis, because they had not yet had an ASD evaluation to rule out the possibility of an ASD diagnosis. Without these cases, the adjusted total sample size is 202 cases and the non-ASD condition consists of 81 cases.

^*∗∗∗*^ *p* < .001.
